# Disease Animal Models of TDP-43 Proteinopathy and Their Pre-Clinical Applications

**DOI:** 10.3390/ijms141020079

**Published:** 2013-10-09

**Authors:** Yu-Chih Liu, Po-Min Chiang, Kuen-Jer Tsai

**Affiliations:** 1 Institute of Clinical Medicine, National Cheng Kung University, Tainan 704, Taiwan; E-Mails: s96981012@mail.ncku.edu.tw (Y.-C.L.); pmchiang@mail.ncku.edu.tw (P.-M.C.); 2 Institute of Basic Medical Science, National Cheng Kung University, Tainan 704, Taiwan

**Keywords:** amyotrophic lateral sclerosis, disease models, frontotemperal lobar degeneration, proteinopathy, TDP-43, therapy

## Abstract

Frontotemperal lobar degeneration (FTLD) and amyotrophic lateral sclerosis (ALS) are two common neurodegenerative diseases. TDP-43 is considered to be a major disease protein in FTLD/ALS, but it’s exact role in the pathogenesis and the effective treatments remains unknown. To address this question and to determine a potential treatment for FTLD/ALS, the disease animal models of TDP-43 proteinopathy have been established. TDP-43 proteinopathy is the histologic feature of FTLD/ALS and is associated with disease progression. Studies on the disease animal models with TDP-43 proteinopathy and their pre-clinical applications are reviewed and summarized. Through these disease animal models, parts of TDP-43 functions in physiological and pathological conditions will be better understood and possible treatments for FTLD/ALS with TDP-43 proteinopathy may be identified for possible clinical applications in the future.

## 1. Introduction

With the advent of medical and technical advances, people may now live longer. Age-related diseases also gain more attention, especially neurodegenerative diseases. A common feature of such neurodegenerative diseases is the misfolding and aggregated disease protein in the central nervous system of patients. A typical example is the transactive response DNA-binding protein (TDP-43), which plays a pivotal role in the pathogenesis of frontotemporal lobar degeneration (FTLD) and amyotrophic lateral sclerosis (ALS). A common neurodegenerative disease, FTLD accounts for 5%–24% of early-onset dementia. Patients are characterized by marked atrophy in the frontal and temporal lobes of the brain and have behavior and language dysfunctions [1,2]. Some of FTLD patients also have abnormal motor symptoms similar to motor neuron diseases (MND).

Moreover, 15% of the patients match the diagnostic criteria of ALS [3], which is a type of MND with selective neuronal degeneration in both upper and lower motor neurons. Patients with ALS show progressive muscle atrophy, weakness, and spasticity. The median survival from the onset of ALS is 3–5 years. More importantly, a certain degree of frontal dysfunction can be observed in 47% of patients. Those with co-morbid ALS and FTLD are referred to as FTLD-ALS and these patients only have an average survival of 2 years after diagnosis. The co-morbidity implies a common pathologic mechanism between these two diseases [3,4].

**Figure 1:**
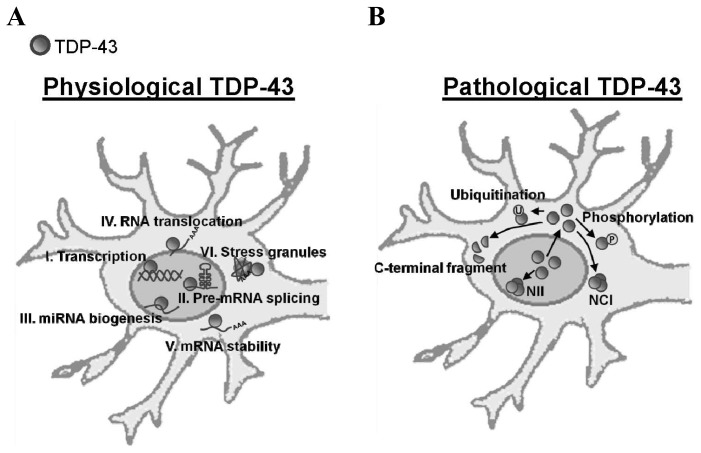
Expression of TDP-43 in physiological and pathological conditions. The **upper panel** shows the physiological functions of TDP-43, including transcription, pre-mRNA splicing, mRNA biogenesis, stress granules formation and mRNA stability. The **lower panel** shows the pathological expressions of TDP-43, which is predominantly sequestered in the cytoplasm, different from TDP-43 proteinopathy. (**A**) The roles of TDP-43 in the physiological neuron; (**B**) The morphologies of TDP-43 in the pathological neuron.

Since 2006, TDP-43 has been recognized as the major disease protein in FTLD and ALS [5]. It is a 43 kDa protein predominantly located in the nuclei. It has two RNA-recognition motifs (RRM) that allows it to bind to DNA and RNA, and a glycine-rich *C*-terminal domain in TDP-43, which allows it to bind to proteins. As a member of the heterogeneous nuclear ribonucleoprotein complex (hnRNP), TDP-43 has functions in transcription, RNA processing, microRNA biogenesis and RNA splicing [6–9]. It also targets about 6000 genes [10]. Furthermore, a small portion of TDP-43 is expressed in the cytosol, where it may be involved in stress granular formation and mRNA stability (Figure 1A) [11,12].

**Figure 2:**
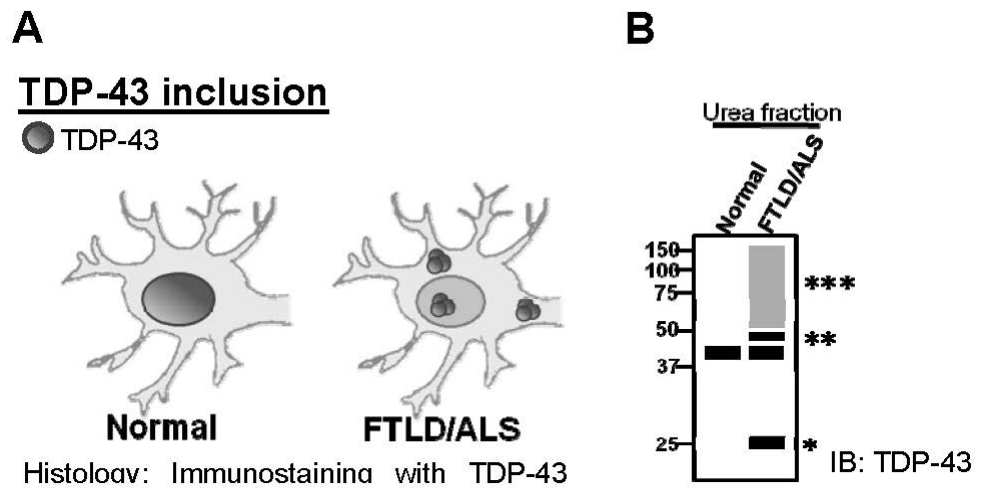

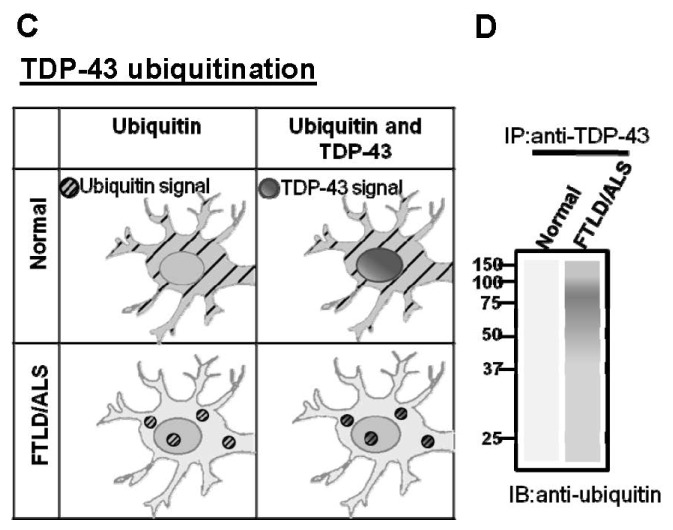
Experiments to examine TDP-43 proteinopathy in diseases animal models. (**A**) In histological staining with TDP-43 antibody, physiological TDP-43 is predominantly expressed in the nucleus of neurons, whereas in FTLD/ALS patients, TDP-43 would form inclusion bodies in the cytoplasm (NCI) or nuclear (NII); (**B**) Truncated TDP-43, phosphorylated TDP-43 and ubiquitinated TDP-43 is expressed in TDP-43 inclusions; (**C**) Through co-immunostaining with TDP-43 and ubiquitin antibody, signals of ubiquitin and TDP-43 would be detected in different subcellular localizations. However in FTLD/ALS, TDP-43 would co-localize with ubiquitin in the nucleus to form NII or in the cytoplasm to form NCI; (**D**) TDP-43 is tightly associated with ubiquitin in the CNS of FTLD/ALS by co-immunoprecipitation. * truncated TDP-43 (25 kDa); ** phosphorylated TDP-43; *** high molecular TDP-43 (ubiquitinated TDP-43).

However, in pathologic conditions such as FTLD and ALS, TDP-43 is expressed in cytoplasmic ubiquitin inclusions that are accompanied by nuclear clearance of “normal” TDP-43 or form neuronal intranuclear inclusions (NII) in the central nervous system (CNS) (Figure 1B). TDP-43 is abnormally processed, including ubiquitination, phosphorylation and *C*-terminal truncation, when it is sequestered to the cytosol. To discover the pathologic link between TDP-43 and FTLD/ALS as well as potential therapy, animal models with TDP-43 abnormal expression have been established. These models enable the examination of the hallmarks of TDP-43 proteinopathy in the CNS of disease animal models (Figure 2A–D), including TDP-43 inclusion, which can be examined by urea fraction, and TDP-43 ubiquitination, which can also be checked by co-immunoprecipitation.

This review summarizes TDP-43 proteinopathy in recently developed disease animal models with abnormal TDP-43 expression and reviews recent advances in potential therapy using these animal models.

## 2. Disease Animal Models of TDP-43 Proteinopathy: Mammalian Models

### 2.1. Wild-Type or Mutant TDP-43 Overexpression by Transgenic Animals

In FTLD/ALS, the expression of TDP-43 is around 1.5 fold higher than that in healthy individuals [13,14]. To discover the pathologic roles of TDP-43, some transgenic mammals that overexpress WT human or mouse TDP-43 have been developed. Aside from higher TDP-43 level, some consistent mutations have also been discovered in familial FTLD/ALS. Another strategy is the creation of the FTLD/ALS disease mammals via expressing mutant TDP-43. Either WT or mutant TDP-43 overexpression often promotes TDP-43 gain of toxic functions, resulting in neurodegeneration.

To gain insights into the role of TDP-43 in specific neurons or tissue, specific promoters have been provided to drive exogenous TDP-43 overexpression. Overexpression of WT or mutant TDP-43 under the control of different promoters trigger different expression pattern of exogenous TDP-43 and lead to transgenic mammals with different histological features and phenotypes. The TDP-43 proteinopathy and phenotypes of these transgenic mammalian models are summarized in Table 1. The following section shows the classification of these mammalian models by promoters.

#### 2.1.1. Mouse Prion Promoter

Mouse prion promoter is a common promoter used to drive neurodegeneration associated disease protein expression in transgenic mice. Transgenes controlled by the mouse prion promoter express high levels of the protein in the CNS and heart. This may extend to other tissues such as skeletal muscle, the lungs and liver. Some groups generate the transgenic mice overexpressing WT-hTDP-43 or mutant hTDP-43 driven by the mouse prion promoter [15–19]. According to their reports, overexpression of WT or mutant hTDP-43 induced motor dysfunction, such as gait disorders. Xu *et al*. report that overexpression of WT-hTDP-43 is sufficient to trigger mice with motor deficits and TDP-43 proteinopathy in the CNS of transgenic mice [17]. In their study, moderate expression of hTDP-43 (about 2.5 fold) can promote cytoplasmic TDP-43 expression, TDP-43 truncation, TDP-43 phosphorylation and TDP-43 aggregation, which mimics the TDP-43 proteinopathy in CNS of FTLD/ALS. Besides TDP-43 proteinopathy, abnormal mitochondrial aggregation is observed in hTDP-43-overexpressing transgenic mice, indicating the involvement of TDP-43 in mitochondrial trafficking.

#### 2.1.2. Murine Thy-1.2 Promoter

Murine Thy-1.2 promoter (mThy1.2) can drive transgene expression postnatally in all neurons of the CNS. Some groups overexpress the WT or mutant hTDP-43 under the control of mThy1.2 to trigger transgenic mice with FTLD/ALS-like symptoms [20–22]. These studies show that the postnatal TDP-43 overexpression in all neurons results in neuronal intranuclear inclusions (NII) and neuronal cytoplasmic inclusions (NCI) formation, and the generation of truncated TDP-43. Abnormal distribution of Gemini of coiled bodies (GEMs) and mitochondria accumulation are also identified in the motor neurons of TDP-43 overexpressing transgenic mice, even without TDP-43 cytoplasmic inclusions [21].

In addition, studies also reveal that WT-hTDP-43 overexpression in neurons is sufficient to trigger motor dysfunction in mice, suggesting that such overexpression may be pathogenic in FTLD/ALS. The truncated TDP-43 (~25 KDa) has been involved in disease progression by gain of toxic function [20]. Thus TDP-25 has also been introduced to create the transgenic mouse model [23]. Transgenic mice expressing the 25 kDa *C*-terminal fragment of TDP-43 develop cognitive deficits without TDP-43 inclusions and trigger the processing of endogenous TDP-43, which suggest that TDP-43 inclusions are independent of cognitive dysfunction.

#### 2.1.3. CamKII Promoter

Calmodulin-dependent protein kinase II (CamKII) is an important kinase in learning/memory and is highly expresses in the forebrain area. Overexpression of WT mouse TDP-43 (mTDP-43) under the control of mouse CamKII promoter increases mTDP-43 expression in the forebrain of transgenic mice [24]. mTDP-43 overexpression in the hippocampus and cortex induced impaired cognitive function in the 2 month-old and motor dysfunction in the 6 month-old transgenic mice. It is the first article to check both cognitive functions and motor functions of transgenic mice with TDP-43 overexpression. In addition to phenotypes, this transgenic mouse has similar TDP-43 proteinopathy with FTLD/ALS patients. Overexpression of mTDP-43 in mouse forebrain not only induced cytoplasmic TDP-43 inclusion but also lose “normal” TDP-43 in the nuclei. Moreover, mTDP-43 in the forebrain only is sufficient to trigger motor dysfunction in mice. Transgenic mouse with mTDP-43 overexpression in forebrain may be the ideal disease model for addressing the important problem on the pathologic link between FTLD and MND.

#### 2.1.4. CamKII Promoter Combined with Tet-off System

Both FTLD and ALS are age-related neurodegenerative diseases and patients are predominantly middle-aged. Inducible transgenic mice with the WT-hTDP-43 or NLS (nuclear localization signal) mutant hTDP-43overexpression under the control of the CamKII promoter have been generated to characterize the pathologic roles of TDP-43 in different developmental periods [25,28]. Mice expressing hTDP-43 in the forebrain after sexual maturation developed TDP-43 proteinopathy in the CNS and showed motor dysfunction. In contrast, the transgenic mice with NLS mutant hTDP-43 expression have more severe motor deficits.

Furthermore, expressing hTDP-43 with defective NLS in the forebrain not only results in MND symptoms but in the overexpression of cytoplasmic TDP-43 and a reduction in endogenous mTDP-43. However, rare cytoplasmic TDP-43 inclusions exist in the forebrain of inducible transgenic mice with WT-hTDP-43 or NLS mutant hTDP-43 overexpression, indicating that cytoplasmic inclusions might not be required for neurodegeneration [25]. Thus dysregulation of TDP-43 may have more severe effects in early brain development than in mature brain [28].

#### 2.1.5. Human Endogenous Promoter

To fully mimic the FTLD/ALS human patients, the human TDP-43 endogenous promoter has been used to drive WT or mutant hTDP-43 overexpression in transgenic mice [27]. Mutant TDP-43 (A315T or G348C) overexpression under the control of human TDP-43 endogenous promoter induces TDP-43 aggregation, cytoplasmic TDP-43 overexpression and truncated TDP-43 expression in CNS of Transgenic mice. The overexpression of WT or mutated TDP-43 manifests with cognitive and motor dysfunctions during aging, which mimics the course observed in FTLD/ALS patients. Abnormal TDP-43 ubiquitous expression shows intermediate filament abnormalities and axonatrophy. Apart from transgenic mouse model, transgenic rat models have also been created to reproduce the clinical phenotype of FTLD/ALS patients and help find the possible pathologic mechanism and potential therapy.

The overexpression of WT-hTDP-43 or mutant hTDP-43 under the control of human endogenous promoter in rat increases cytoplasmic TDP-43 expression [29]. However, only the rats with mutant hTDP-43 (M337V-hTDP-43) expression exhibited significant motor dysfunction, which is different from other mouse models. M337V-hTDP-43 expression in rat also reproduces the biochemical features of FTLD/ALS including hyperphosphorylation of TDP-43, formation of TDP-43 inclusion and expression of truncated TDP-43. Most importantly, the ubiquitous expression of M337V-hTDP-43 in rat is seen to cause more severe neurodegeneration in the motor system than in the CNS.

#### 2.1.6. Others

The CAG promoter has also been used to drive WT or mutant hTDP-43 ubiquitous expression in transgenic mammals. Transgenic mice with WT-hTDP-43 overexpression under the control of CAG promoter displayed only moderate loss of cortical neurons without any significant FTLD/ALS phenotype or TDP-43 proteinopathy [26]. M337V-hTDP-43 expression driven by CAG promoter resulted however in transgenic rats with motor dysfunction and elevated level of cytoplasmic TDP-43 and truncated TDP-43 [29]. In addition, the promoter of human neurofilament heavy chain (NEF) and choline acetyltransferase (ChAT) also have been chosen to direct mutant hTDP-43 expression in transgenic rat models. Inducible transgenic rats with M337V-hTDP-43 expression have been designed to help identify the pathologic roles of TDP-43 in mature brain [30]. Transgenic rat with mutant hTDP-43 expression under the control of the ChAT promoter display motor dysfunction and cytoplasmic TDP-43 aggregation, the phenotype and histologic features of ALS patients. This indicates that M337V-hTDP-43 expression in motor neurons alone is sufficient to trigger the onset of ALS. Intriguingly, suppressing M337V-hTDP-43 expression by withdraw doxycyclin in inducible transgenic rats can partially reverse disease progression.

#### 2.1.7. Brief Summary of Transgenic Mammals

Different promoters trigger WT or mutant TDP-43 overexpression in different tissues and result in transgenic mammals with different pathological and biochemical features. Prion promoter, human endogenous promoter and CAG promoter drive widespread overexpression of WT or mutant TDP-43 rather than neuron specific expression in transgenic mammals. The prion promoter is the first promoter used to generate the TDP-43 transgenic mammal. However, WT or mutant TDP-43 overexpression under the control of the prion promoter often causes transgenic mammals with early lethality. Overepxression of WT or mutant TDP-43 regulated by a human endogenous promoter may be the better disease mammal models, which may cause transgenic mice with age-related neurodegeneration and motor dysfunction mimicked FTLD/ALS patients. However, only mutant TDP-43 overexpression under the control of human endogenous promoter triggers transgenic mammal with TDP-43 proteinopathy like FTLD/ALS. To emphasize the toxic functions of TDP-43 in neurons, the Thy1.2 promoter, CaMKII promoter, NEF promoter and ChAT promoter were chosen to regulate WT or mutant TDP-43 overexpression in neurons. There are some differences between these promoters, including (1) expression time-point: the Thy1.2 promoter regulates WT or mutant TDP-43 overexpression in the postnatal stage; (2) specific neuron population: the CaMKII promoter triggers WT or mutant TDP-43 overexpression in forebrain, the ChAT promoter promotes overexpression of WT or mutant TDP-43 in motor neuron. WT mTDP-43 overexpression under the control of the CaMKII promoter not only displays cognitive dysfunction but also motor impairment. Most importantly, as in human FTLD-MND patients, CaMKII-TDP-43 Tg mice exhibit cognitive dysfunction at an early disease stage (2-months old), which is followed by motor dysfunction (6-months old). Further, the tet-off system has been taken advantage of to generate the transgenic mammal for control of the expression time of transgenes. Induction of NLS mutant hTDP-43 overexpression after sexual maturation in forebrain of mice leads to progressive neuronal loss; cerebral atrophy and motor spasticity, which mimics human FTLD/ALS patients. No matter which promoters were used to generate the transgenic mammal with TDP-43 proteinopathy, some common features have been discovered and might be involved in the FTLD/ALS progression. Mitochondria dysfunction, abnormal distribution of GEM, neurofilament abnormalities and neuroinflammation are some pathological features in transgenic mammal with TDP-43 proteinopathy and may be some critical events for disease progression. According to the studies on transgenic mammal with TDP-43 proteinopathy, different neuron populations would display different sensitivities to dysregulation of TDP-43. In CNS, pyramidal neurons in layer five and spinal cord neurons are more sensitive to abnormal TDP-43 expression. Besides, overexpression of WT or mutant TDP-43 in motor neurons is seen to have profound effects and sufficient to cause transgenic mammals with ALS symptoms. Furthermore, TDP-43 inclusion may not be required for neurodegeneration. In contrast, redistribution of TDP-43 and altering of RNA/DNA binding affinity or RNA splicing may play important roles in disease progression.

### 2.2. Wild-Type or Mutant TDP-43 Overexpression by Virus Induced System

Apart from the constitutive conditions of transgenic animals, virus-induced systems are another way of driving WT or mutant TDP-43 overexpression. Such methods will be simple and fast, but the delivery system does not show the long-term expression of both WT and mutant TDP-43 in animals. Some mammalian models of TDP-43 proteinopathy established by viral transduction are shown in Table 2.

#### 2.2.1. Non-Human Primate Model

To minimize species differences, non-human primate models of TDP-43 proteinopathy have been created. Cynomolgus monkeys were injected with the adeno-associated virus (AAV) vector and overexpressed WT-hTDP-43 in the spinal cord [31]. Overexpression of WT-hTDP-43 in monkeys promote the up-regulation of cytoplasmic TDP-43, phosphorylated TDP-43 and TDP-43 aggregation. These monkeys developed progressive motor weakness and muscle atrophy. However, there is no truncated TDP-43 expressed in the monkey model, which indicates that TDP-43 truncation may not be required for motor neurodegeneration. Phosphorylated TDP-43 occurs after motor dysfunction in the monkey model. Lastly, the report showed that mislocalization of TDP-43 may be the major contributor to the degeneration of motor neurons rather than other TDP-43 proteinopathy.

#### 2.2.2. Rat Models

Some rat models with TDP-43 proteinopathy have been established by virus delivery. Overexpression of the WT-hTDP-43 in the spinal cord, substantia nigra (SN), or hippocampus of rat by injecting AAV induces different phenotypes and TDP-43 proteinopathy of FTLD/ALS [31–34]. Rats with WT-hTDP-43 overexpression in the spinal cord or SN display motor dysfunction, whereas overexpression in the hippocampus causes cognitive deficits. Widespread overexpression of WT-hTDP-43 in rat by intravenous AAV injection leads to severe motor symptoms like ALS and ~50% survival rate within four weeks after injection [35]. Rats with WT-hTDP-43 overexpression in the motor cortex have also been created by lentiviral injection [36]. Such overexpression in motor cortex of rats triggered TDP-43 aggregation, up-regulation of cytoplasmic TDP-43, truncated TDP-43 and phosphorylated TDP-43. It also induces the production of the amyloid precursor protein (APP) *C*-terminal fragment and enhances the activity of β-secretase (BACE), suggesting that TDP-43 is involved in the metabolism of APP. Viral injection of mutant hTDP-43 has been conducted to induce TDP-43 proteinopathy in rats [34]. Overexpression of hTDP-43 with the 25 kDa *C*-terminal of TDP-43 (hTDP-25) or NLS mutant TDP-43 in rats causes rotarod deficits and hindlimb paresis. The motor dysfunctions mimic those of ALS. The overexpression of hTDP-25 results in selective forelimb impairment in rats, suggesting that the short fragment of TDP-43 may be involved in the disease progression.

#### 2.2.3. Brief Summary of Viral-Modeling Mammalian Models

Compared to transgenic mammals, the vector-modeling system is a shorter and more cost-effective strategy for modeling neurodegenerative diseases. The virus approach can also easily co-express two transgenes and may be useful for studies on the interaction between genes. Moreover, one of the hypotheses about the progression of TDP-43-related neurodegenerative diseases is the “two-hit” mechanism and the virus-based system may be useful for examining the “two-hit” hypothesis [37]. However, the virus delivery system is less stable, cannot trigger the long-term effect of transgenes and is restricted by viral infection. Mammals overexpressing WT-hTDP-43 via virus transduction is sufficient to have pathologic features like ALS patients. The phenotypes of monkey models with TDP-43 proteinopathy might be more close to human patients than rodent models. Monkeys with hTDP-43 overexpression displayed progressive motor weakness, muscle atrophy and morphological features like type B TDP-43 proteinopathy, which mimics human ALS patients. Therefore, the monkey models of TDP-43 proteinopathy may be valuable disease models for studying the pathogenesis of ALS. Though aberrant expression of human TDP-43 did not trigger TDP-43 aggregation in rodent models of TDP-43 proteinopathy, these rat models still exhibited motor dysfunction. These results indicated that TDP-43 inclusion might not be required for the onset of FTLD/ALS.

### 2.3. TDP-43 Knockout Animals

#### 2.3.1. TDP-43 Knockout Mammalian Models

The pathologic mechanism of FTLD/ALS resulting from the loss of TDP-43 function is another important issue to deal with. Some mammalian models with loss of TDP-43 have shown that TDP-43 is important for embryonic development (Table 3). Mice with TDP-43 knockout results in embryonic lethality [38–40]. To avoid this, mice with a conditional knockout of TDP-43 have been generated [41]. Interestingly, such mice show significant loss of body weight but without significant FTLD/ALS symptoms, indicating that TDP-43 may also be involved in the regulation of body-fat metabolism.

On the other hand, targeted depletion of TDP-43 in the spinal cord motor neurons of mice induces progressive motor dysfunction and muscle weakness/atrophy, the ALS-related syndromes [42,43]. Aside from the pathologic feature, loss of TDP-43 in motor neurons also leads to ubiquitin aggregation, which implies that loss of TDP-43 function may be a major cause of ALS with TDP-43 proteinopathy.

#### 2.3.2. Brief Summary of Knockout Mammalian Models

The major functions of TDP-43 include transcriptional regulation and RNA splicing. These functions need the nuclear “normal” TDP-43. However, normal TDP-43 signals are absent in CNS neurons of FTLD/ALS human patients. To investigate the linkage between the loss-of-function of TDP-43 and pathological features FTLD/ALS, TDP-43 knockout models have been created. TDP-43 is important for early embryonic development; TDP-43 knockout in mice would result in peri-implantation lethality. Targeted depletion of TDP-43 in motor neurons could trigger mice with age-dependent progressive motor neuron degeneration, muscle atrophy and motor dysfunction, which are reminiscent of human ALS patients. These findings indicated that TDP-43 might be important for the long-term maintenance of motor neuron. Therefore, the mouse model with depletion of TDP-43 expression in motor neuron not only has a longer lifespan compared to the whole body knockout, but also could be a valuable model for studying the role of TDP-43 in motor neurons.

## 3. Disease Animal Models of TDP-43 Proteinopathy: Non-Mammalian Models

### 3.1. Wild-Type or Mutant TDP-43 Expression by Transgenic Animals

#### 3.1.1. Wild-Type or Mutant TDP-43 Expressed in *C. elegans*

Apart from mammalian models, non-mammalian models with overexpression of the WT or mutant TDP-43 have been created to discover the pathologic roles of TDP-43 in FTLD/ALS (Table 4). *Caenorhabditis elegans* (*C. elegans*) is a common model organism for generating a convenient disease animal model. Overexpression of either WT-TDP-1 (*C. elegans* ortholog of TDP-43) or hTDP-43 in worms causes an uncoordinated phenotype [44]. Transgenic worms expressing hTDP-43 have neurotoxicity without obvious TDP-43 proteinopathy. However, another study states that a transgenic worm develops TDP-43 proteinopathy by expressing WT-hTDP-43 [45,46] and that the *C. elegans* models with the WT or mutant (A315T, G290A, M337V, Q331K and C25) hTDP-43 expression captures some characteristics of FTLD/ALS, including TDP-43 aggregation, truncation and phosphorylation. Most importantly, these transgenic worms with TDP-43 proteinopathy display age-dependent motor dysfunction, mimicking human patients.

Furthermore, insulin/insulin-growth factor 1 (IGF-1) signaling may be a therapeutic target, because deficiency in insulin/IGF-1 signaling can alter the neurotoxicity and protein aggregation that results from hTDP-43 expression. In addition to pan-expression of WT or mutant hTDP-43, transgenic worms with specific neuronal expression of hTDP-43 have been created [47]. However, only transgenic worms with A315T-hTDP-43 expression show age-dependent motor impairment and TDP-43 inclusions. Based on these results, transgenic worms with TDP-43 proteinopathy can be seen as a simple and convenient disease model for developing potential therapy and drug screening.

#### 3.1.2. Wild-Type or Mutant TDP-43 Expressed in Zebrafish

The ubiquitous expression of mutant (A315T**,** G348C and A382T) hTDP-43 in zebrafish causes shorter motor axons, premature, excessive branching and swimming deficits. These imply that the mutant hTDP-43 may gain toxic functions that contribute to the pathogenesis of ALS [48]. Expression of mutant TDP-43 with a premature stop codon (Y220X) in zebrafish eliminates the expression of TDP-43 and causes shorter motor axons, locomotor deficits and early death [49]. Transgenic zebrafish with Y220X TDP-43 expression may therefore be a kind of *tardbpl* knockout model that can be used for studies on the functional loss of TDP-43 in the pathogenesis of FTLD/ALS.

#### 3.1.3. Wild-Type or Mutant TDP-43 Expressed in Sensory Neuron of Drosophila

Drosophila is another model organism used to generate transgenic models of TDP-43 proteinopathy. The UAS/GAL4 system can be used to trigger the expression of the transgene in a specific site of Drosophila. For example, combining UAS/GAL4 with the GMR promoter can drive the transgene to express in the eyes of Drosophila.

Overexpression of Drosophila TDP-43 (dTDP-43) or WT-hTDP-43 in the sensory neuron of Drosophila by using GAL4^211^ shows increased dendrite branching, which is attenuated by the overexpression of the mutant hTDP-43 [49]. TDP-43 regulates the dendritic structural integrity. Some groups have generated transgenic Drosophila models that overexpress WT or mutant TDP-43 in the eyes [50–53,56,57], resulting in the up-regulation of cytoplasmic TDP-43 and the formation of TDP-43 inclusion [50]. On the other hand, overexpression of mutant hTDP-43, such as NLS-mutant hTDP-43, in Drosophila eyes triggers increased cytoplasmic TDP-43 and phosphorylated TDP-43 [56]. Expression of T202-hTDP-43 (which lacks the *N*-terminal RNA recognition motif), in the eyes does not lead to significant retinal degeneration in transgenic flies. These findings indicate that the overexpression of hTDP-43, but not the *C*-terminal fragment alone, in Drosophila eyes causes similar TDP-43 proteinopathy as that in FTLD/ALS in human.

#### 3.1.4. Wild-Type or Mutant TDP-43 Expressed in Motor Neuron of Drosophila

ALS is a common motor neuron disease that results in upper or lower motor neuron degeneration. To address the role of TDP-43 in the pathogenesis of ALS, transgenic Drosophila with WT or mutant TDP-43 expression in motor neurons have been generated [51,52,54,55,57,58,60,62]. The WT-hTDP-43 expression under the control of OK371 causes Drosophila with TDP-43 inclusions, up-regulation of cytoplasmic TDP-43, and motor dysfunction similar to that found in ALS patients [51]. Furthermore, WT or A315T-hTDP-43 overexpression, driven by D42 leads to motor deficits, implying that elevated levels of TDP-43 may have toxic effects in ALS. On the other hand, the overexpression of A315T-hTDP-43 in Drosophila causes more severe locomotor dysfunction than WT-hTDP-43 overexpression, suggesting that studies of individual mutations of TDP-43 are required to elucidate the toxic functions of TDP-43 in FTLD/ALS.

#### 3.1.5. Wild-Type or Mutant TDP-43 Expressed in Pan-Neuronal or Specific Neuron of Drosophila

The elav promoter is a common promoter used for triggering the pan-neuronal expression of transgenes in Drosophila. The WT-dTDP-43 or hTDP-43 overexpression under the control of elav causes the cleavage of TDP-43 and the elevation of both cytoplasmic TDP-43 and phosphorylated TDP-43 [55,56,59,62]. To clarify the differences between disease-associated TDP-43 mutations, Drosophila that overexpress various kinds of mutant TDP-43 have been created. However, transgenic Drosophilae with disease-associated mutations do not display significant TDP-43 proteinopathy. Interestingly, the overexpression of the *C*-terminal fragment of hTDP-43 with mutant phosphorylation sites, which results in the elimination of TDP-43 phosphorylation, leads to TDP-43 aggregation and the up-regulation of cytoplasmic TDP-43 [59]. In contrast, mutant hTDP-43 can trigger TDP-43 phosphorylation that reduces TDP-43 inclusion. This report indicated that hyperphosphorylated TDP-43 may be involved in reducing TDP-43 aggregation.

Furthermore, the overexpression of WT dTDP-43 under the control of the OK137 promoter triggers the up-regulation of cytoplasmic TDP-43 and the formation of TDP-43 inclusion in mushroom bodies [58], which leads to cognitive deficits. Thus, abnormal expression of TDP-43 in Drosophila can affect cognitive function and trigger a phenotype similar to human FTLD patients. Other transgenic Drosophila with WT-hTDP-43 overexpression in specific neurons, such as the CCAP neuron and upper motor neuron, have also been established to clarify the toxic function of TDP-43 in the molecular mechanism of FTLD/ALS [61,62]. The overexpression of dTDP-43 in CCAP neurons can trigger intranuclear and cytoplamic TDP-43 inclusion and wing inflation defects in Drosophila. This also alters the transcriptome and increases the expression of microtubule-associated protein similar to the down-regulation of TDP-43. Therefore, the neurotoxicity of TDP-43 in disease progression may be due to the loss of normal TDP-43 function.

#### 3.1.6. Brief Summary of Non-Mammalian Transgenic Animal Models

Non-mammalian transgenic animal models of TDP-43 proteinopathy are common and convenient disease models for discovering the pathological roles of TDP-43 in FTLD/ALS. *C. elegans* and zebrafish have simple neuron systems and could provide convenient models for large-scale drug screening and outcome evaluation. Transgenic *C. elegans* expressing A315T-hTDP-43 or a high level of WT-hTDP-43 would lead to age-related motor dysfunction, shorter lifespan and TDP-43 proteinopathy. Specific expression of A315T-hTDP-43 in GABAergic neuron also causes *C. elegans* with ALS-like features, including TDP-43 inclusion and motor dysfunction. Such transgenic *C. elegans* models can be useful for initial drug screening. Expression of mutant TDP-43 in zebrafish causes swimming defects, implying TDP-43 is important for motor functional maintenance.

In addition to *C. elegans* and zebrafish models, transgenic Drosophilae of TDP-43 proteinopathy have also been generated. Neurotoxicity due to abnormal TDP-43 levels caused by overexpression of either WT or mutant hTDP-43 may be easily evaluated in Drosophila eyes. For instance, use of toluidine blue staining can examine the eye structure of Drosophila to evaluate the toxic effects of aberrant TDP-43 expression. However, Drosophila eyes with abnormal TDP-43 expression can not function with the motor neurodegeneration. Specific overexpression of either WT or mutant TDP-43 in motor neurons of Drosophilae leads to progressive motor dysfunction and some of them with up-regulation of cytoplasmic TDP-43. Pan-neuronal overexpression of mutant TDP-43 in Drosophila not only causes up-regulation of cytoplasmic TDP-43 but hyperphosphorylated TDP-43.

Interestingly, the study on Drosophila expressing the mutant CTF of TDP-43 shows that hyperphosphorylated TDP-43 may against the formation of TDP-43 inclusion. However in studies on other models of TDP-43 proteinopathy, hyperphosphorylation has still been considered to promote FTLD/ALS progression. More studies are needed for validating the role of phosphorylated TDP-43 in the disease progression. According to these findings, non-mammalian transgenic models of TDP-43 proteinopathy are easy manipulated and convenient models for indentification of the pathological roles of TDP-43 and potential drug discovery.

### 3.2. TDP-43 Knockout Model

#### 3.2.1. TDP-43 Knockout Non-Mammalian Models

Non-mammalian models with loss of TDP-43 function have also been generated (Table 5). In *C. elegans*, there are two mutant allels of *tdp-1* (*ok803* and *ok781*) that cause a null mutant [63,64]. ok803 and ok781 lack the two RNA reorganization motifs (RRM) and the nuclear export signal (NES) that lead to the functional loss of *tdp-1*, which in turn triggers lower fertility, defects in growth and impaired locomotion. Deletions of mutant *tdp-1* may extend the lifespan and alter defects in transgenic worms with hTDP-43 overexpression. The TDP-43 can modulate lifespan by regulation of protein homeostasis, stress signaling and aging.

In zebrafish, using the AMO sequence targeted to *tardbp* or *tardbpl* gene would result in motor dysfunction as in ALS patients [48,49]. However, in another report, knockout of *tardbp* or *tardbpl* by genome editing with zinc finger nucleases do not trigger motor dysfunction in zebrafish. Only when *tardbp* and *tardbpl* are both doubly knocked-out in zebrafish is there muscle degeneration and significant motor deficits as in ALS [49,65].

Drosophila with loss of TDP-43 may result in high mortality in the embryonic stage such that few survive into the adult stage. Even if TDP-43 knockout Drosophilae survive into the adult stage, survivors develop severe motor dysfunction similar to those in ALS [58,62,66,67]. According to reports, TDP-43 plays an important role in the development of motor neurons.

#### 3.2.2. Brief Summary of TDP-43 Knockout Non-Mammalian Models

Knockout of TDP-43 in mammalian models might cause embryonic lethality, though in fact, most TDP-43 knockout non-mammalian models did not cause embryonic lethality and *tdp-1* knockout in *C. elegans* even has the longer lifespan. Though loss of TDP-43 in non-mammalian models might not cause embryonic lethality, these non-mammalian models still have some ALS-like deficits, such as motor dysfunction. The neuron system in non-mammalian animals might be simpler than mammals. But knockout of TDP-43 still caused impaired motor function, which indicated the importance of TDP-43 in the motor neurons. Moreover, the susceptibility to TDP-43 neurotoxicity in motor neurons is seen to be different from other neuron populations. Further studies are needed to validate this interesting problem.

## 4. Recent Advances in Therapy

**Figure 3:**
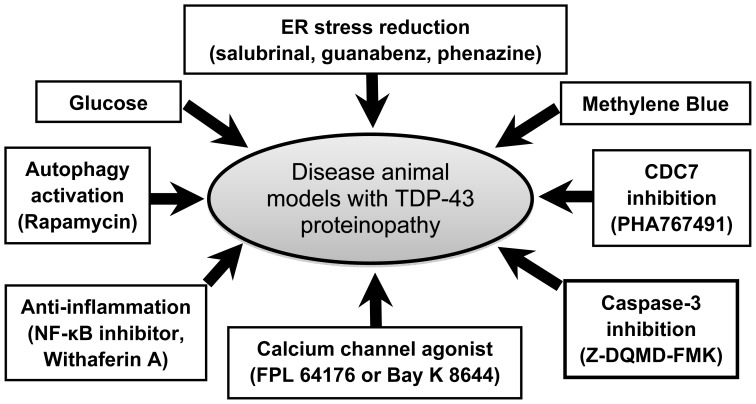
The potential treatments for FTLD/ALS with TDP-43 proteinopathy. Four potential therapeutic strategies for FTLD/ALS with TDP-43 proteinopathy were identified through disease animal models with TDP-43 proteinopathy.

Through various disease models, some potential strategies have been discovered that may be applicable to the clinical treatment of FTLD/ALS. These potential therapeutic strategies include glucose enhancement [68], methylene blue administration [69,70], ER stress reduction [71], CDC7 inhibition [72], anti-inflammation [73], autophagy activation [74,75], calcium channel activation [76] and caspase-3 inhibition [77] (Figure 3). In the following sections, we will introduce these potential strategies, their therapeutic targets and effects on disease animal models of TDP-43 proteinopathy.

### 4.1. Glucose Enhancement

Caloric restriction is a measure of limited dietary intake that has been reported to have positive effects on healthy life and lifespan extension [78]. However, in the transgenic worm expressing A315T-hTDP-43 in GABAergic neuron, caloric restriction does not ameliorate neuronal proteotoxicity [68]. Instead, glucose enhancement may delay this proteotoxicity. High-level glucose administration can reduce the unfolding protein through the network of chaperone proteins. However, glucose enhancement has a negative effect on lifespan. Transgenic worms expressing A315T-hTDP-43 with excess glucose intake exhibits a lower percentage of paralysis even though glucose enhancement decreases the lifespan. Thus, glucose homeostasis is important for lifespan and glucose supplement in neurons may improve neuronal proteotoxicity.

### 4.2. Methylene Blue Administration

Methylene blue (MB) is a common compound used in diagnostic procedure and treatments for cyanide poisoning, malaria, infection and methemoglobinemia [79–81]. It is known as an electron carrier with neuroprotective effects on various neuronal disorders such as stroke and Parkinson’s disease [82,83]. In transgenic worms expressing A315T-hTDP-43, MB can reduce the paralysis rate, against age-dependent neurodegeneration due to abnormal TDP-43 expression. At the same time, MB incubation improves motor functions and rescues abnormally shortened and branched axonal processes in transgenic zebrafish with G348C-hTDP-43 expression.

According to the non-mammal models of TDP-43 proteinopathy, MB may be neuroprotective against neuronal toxicity by reducing the oxidative stress [69]. However, in transgenic mice expressing G348C-hTDP-43, MB fails to confer protection and MB treatment of transgenic mice does not improve the motor function and TDP-43 proteinopathy [70]. Indeed, the late administration of MB in transgenic worms is less effective in TDP-43 proteinopathy. Therefore, early MB treatment in transgenic mammals with TDP-43 proteinopathy may be a potential therapeutic strategy.

### 4.3. Endoplasmic Reticulum Stress Reduction

The endoplasmic reticulum (ER) is an important organelle for modulating the unfolded protein in cells. Excess levels of unfolded protein can trigger ER stress and the unfolded protein response (UPR). Thus, reduction of the ER stress response may be a potential treatment for FTLD/ALS with TDP-43 proteinopathy. In the non-mammal disease models (*C. elegans* and zebrafish), three compounds can suppress toxicity and reduce paralysis by decreasing the ER stress response [71]. These three compounds are salubrinal, guanabenz and phenazine, and target different branches of the UPR pathway but have one common effect against TDP-43 neuronal toxicity.

### 4.4. CDC7 Inhibition

Blocking TDP-43 phosphorylation may be a potential therapy for patients with TDP-43 proteinopathy. CDC7 (cell division cycle kinase 7) has been identified as the TDP-43 kinase responsible for pathologic TDP-43 phosphorylation in the transgenic worm model [72]. In frontal cortex neurons of FTLD cases, CDC7 are co-expressed with phosphorylated TDP-43. CDC7 may promote pathologic TDP-43 phosphorylation not only in *C. elegans* but also in human. Potential therapy targeting CDC7 has been used in transgenic worm expressing M337V-hTDP-43. The PHA767491 is the ATP-competitive inhibitor of CDC7. In the transgenic worm model of TDP-43 proteinopathy, after treatment with PHA767491, the expression level of phosphorylated TDP-43 is significantly decreased and fewer neurons are lost. Thus, CDC7 inhibition is seen to be a therapeutic strategy for FTLD/ALS.

### 4.5. Anti-Inflammation

Anti-inflammation factor, such as nuclear factor κB (NFκB) inhibitor, is one of the possible treatments for FTLD/ALS with TDP-43 proteinopathy [73]. Gliosis is an important histological feature of such patients [84], who would also have neuroinflammation. The transgenic mouse model with WT or mutant hTDP-43 overexpression also displayed gliosis. Thus, TDP-43 serves as a co-activator of NFκB, an important transcription factor mediating inflammation. The deregulation of TDP-43 triggers NFκB-associating pathogenic mechanism in ALS. TDP-43 transgenic mice treated with Withaferin A (an NFκB inhibitor) eliminated ALS symptoms in mice.

### 4.6. Autophagy Activation

Aside from anti-inflammation, the activation of autophagy is another potential therapy for FTLD/ALS with TDP-43 proteinopathy [74,75,85]. TDP-43 inclusion is the histologic hallmark of FTLD/ALS in the CNS. It is toxic to neurons and leads to neurodegenration. Using autophagy activators such as rapamycin is sufficient to reduce TDP-43 inclusion and improve cognitive and motor deficits in TDP-43 transgenic mice [74,75].

### 4.7. Calcium Channel Activation

The use of calcium channel agonists, like FPL 64176 or Bay K 8644, is also a potential strategy against the motor dysfunctions in ALS with TDP-43 proteinopathy [76]. In zebrafish, impaired swimming and neuromotor dysfunction from the overexpression of mutant hTDP-43 are rescued by chronic treatment with calcium channel agonists. Therefore, treatment with chronic calcium channel agonists can be pursued in mammalian disease models with TDP-43 proteinopathy.

### 4.8. Caspase-3 Inhibition

The TDP-43 proteinopathy is not only expressed in FTLD/ALS but also in other neuropathological conditions, such as Alzheimer’s disease, Parkinson’s disease and stroke [77,86–90]. A previous study found that TDP-43 is cleaved into small *C*-terminal fragments by caspase-3 [91]. Acute ischemic stroke shows increased truncated TDP-43 (25 kDa of TDP-43), decreased expression of full-length TDP-43 and cytoplasmic redistribution of TDP-43 in ischemic penumbra regions of rat. Abnormal TDP-43 expression can be explained by proteolytic cleavage of TDP-43, and application of caspase-3 inhibitor (Z-DQMD-FMK) in rat with acute ischemic stroke reverses this abnormal expression of TDP-43 [77].

To date, effective treatment for FTLD/ALS patients with TDP-43 proteinopathy remains a challenge. Based on models with TDP-43 proteinopathy, researchers aim to understand more about the consequences of abnormal TDP-43 expression in neurons as well as the pathologic functions of TDP-43. Some possible therapeutic strategies may be discovered based on these findings. Thus, there are eight potential treatments. Hopefully these findings can be applied in the clinical setting in the near future.

## 5. Conclusions

More and more groups are engaging in research that examines the role of TDP-43 in the pathogenesis of FTLD/ALS. An increasing number of disease animal models have been set up to provide insights into the molecular mechanism of FTLD/ALS with TDP-43 proteinopathy. Overexpression of WT or mutant TDP-43 in transgenic or inducible models provide evidence of toxic functions and mislocalization of TDP43 in patients with FTLD/ALS. TDP-43 is important for mammalian embryonic development and loss of function causes embryonic lethality. Specific knockout of TDP-43 in motor neurons leads to motor deficits similar to those found in patients with ALS. At the same time, animal models are providing the opportunity to learn and work towards identifying potential therapies for FTLD/ALS. Thus far, there are eight possible treatments that can be utilized. Treatments may soon be applied to the clinical setting. Nonetheless, the exact role of TDP-43 in the pathogenesis of FLTD/ALS still has not been fully unraveled. Further studies on the molecular mechanism of FTLD/ALS with TDP-43 proteinopathy are warranted.

## Figures and Tables

**Table 1:** Mammalian models: wild-type or mutant TDP-43 expression by transgenic animals (NA: non-analysis).

Species	Line	Transgene	Promoter	TDP-43 proteinopathy						Phenotype		Ref.
				TDP-43+ inclusion	ubiquitin+ and TDP-43+ inclusion	Cytoplasmic TDP-43	Loss nuclear TDP-43	Truncated TDP-43 (35 or 25 kDa)	Phosphorylated TDP-43	Cognitive dysfunction	Motor dysfunction	
Mouse	Prp-TDP43^A315T^	Flag- A315T-hTDP-43	mouse prion promoter	X	X	O	O	O	NA	NA	O	[15]
	WT TDP-43 line 21	WT-hTDP-43	mouse prion promoter	X	X	O	NA	O	NA	NA	O	[16]
	A315 TDP-43 line 23	A315T-hTDP-43	mouse prion promoter	O	O (NCI, rare NII)	O	NA	O	O	NA	O	
	M337V TDP-43 line 39	M337V-hTDP-43	mouse prion promoter	NA	NA	O	NA	O	NA	NA	O	
	TDP-43_prp_	WT-hTDP-43	mouse prion promoter	O	O (NCI and NII)	O	NA	O	O	NA	O	[17]
	TDP-43^WT^ TAR4/4	WT-hTDP-43	murine Thy1.2 promoter	O	O (NCI and NII)	O	O	O	O	NA	O	[20]
	TDP-43 Tg W3	WT-hTDP-43	murine Thy1.2 promoter	O	O (NII)	NA	NA	NA	NA	NA	O	[21]
	CaMKII-TDP-43 Tg	WT-mTDP-43	mouse CaMKII promoter	O	O (NCI)	O	O	O	NA	O	O	[24]
	hTDP-43-WT W12	WT-hTDP-43	mouse CamKII- tTA x tet off	O	O (rare NCI and NII)	O	X	X	O	NA	O	[25]
	hTDP-43-ΔNLS ΔNLS4	ΔNLS-hTDP-43	mouse CamKII- tTA x tet off	O	O (NCI)	O	O	X	O	NA	O	
	CAG-TDP-43	WT-hTDP-43	CAG	X	X	X	NA	X	NA	NA	X	[26]
	TDP-43 WT	WT-hTDP-43	human endougenous promoter	X	X	X	X	X	NA	O	O	[27]
	TDP-43 A315T	A315T-hTDP-43	human endougenous promoter	O	O (NCI)	O	O	O	NA	O	O	
	TDP-43 G348C	G348C-hTDP-43	human endougenous promoter	O	O (NCI)	O	O	O	NA	O	O	
	hTDP-43M337V line 4 & 6	M337V-hTDP-43	mouse prion promoter	O (NCI)	X	O	NA	O	O	NA	O	[18]
	TgTDP-25 (B) and (F)	hTDP-25	murine Thy1.2 promoter	X	X	O	NA	O	X	O	NA	[23]
	iTDP-43_WT_ 5a	WT-hTDP-43	mouse CamKII- tTA x tet off	O	O	O	NA	O	O	NA	NA	[28]
	TDP-43^WT^	myc-WT-hTDP-43	mouse prion promoter	X	X	X	NA	NA	NA	NA	X	[19]
	TDP-43^Q331K^	myc-Q331K-hTDP-43	mouse prion promoter	X	X	X	NA	NA	NA	NA	O	
	TDP-43^M337V^	myc-M337V-hTDP-43	mouse prion promoter	X	X	X	NA	NA	NA	NA	O	
	p.M337V-hTDP-43 mt-TAR5/6	M337V-hTDP-43	Thy 1.2	O	O	NA	O	O	O	NA	O	[22]
Rat	miniTDP-43^WT^	WT-hTDP-43	human endougenous promoter	X	X	O	NA	O (35 & 15 kDa)	O	NA	X	[29]
	miniTDP43^M337V^	M337V-hTDP-43	human endougenous promoter	NA	NA	O	NA	NA	NA	NA	O	
	TRE-TDP43^M337V^	M337V-hTDP-43	CAG-tTA x tet off	O	NA	O	NA	O (35 & 15 kDa)	O	NA	O	
	NEF-tTA/TDP-43^M337V^	M337V-hTDP-43	human NEF-tTA x tet off	X	X	NA	NA	NA	NA	NA	O	[30]
	ChAT–tTA-9/TDP-43^M337V^	M337V-hTDP-43	mouse ChAT-tTA x tet off	O	O (NCI)	O	NA	NA	NA	NA	O	

**Table 2:** Mammalian modes: wild-type or mutant TDP-43 overexpression by virus-induced system (NA: non-analysis).

Species		Cynomolgus monkey	Rat						
Virus vector		AAV1	AAV1	AAV9	AAV9	AAV9	Lenti virus	AAV9	AAV9
Delivery gene		Flag- WT- hTDP-43	Flag- WT- hTDP-43	GFP-WT-hTDP43	GFP-WT-hTDP43	GFP-WT-hTDP43	WT-hTDP-43	hTDP-43-ΔNLS	TDP-25
Injection site		Spinal cord (C5–6)	Spinal cord (C6)	Substantia nigra (SN)	Intravenous (1-day-old pup)	Dorsal hippocampus	Motor cortex	Intravenous (1-day-old pup)	Intravenous (1-day-old pup)
TDP-43 proteinopathy	TDP-43+ inclusion	O	X	X	NA	NA	O	X	X
	ubiquitin+ and TDP-43+ inclusion	X	X	X	NA	NA	NA	X	X
	Cytoplasmic TDP-43	O	X	O	NA	NA	O	O	O
	Truncated TDP-43 (35 or 25 kDa)	X	X	NA	NA	NA	O	NA	O
	Phosphorylated TDP-43	O	X	NA	NA	NA	O	X	O
Phenotype	Cognitive dysfunction	NA	NA	NA	NA	O	NA	NA	NA
	Motor dysfunction	O	O	O	O	X	NA	O	O
Ref.		[31]		[32]	[35]	[33]	[36]	[34]	

**Table 3:** Mammalian model: TDP-43 knockout model (NA: non-analysis).

Species	Line	Deletion	Deletion site	Embryonic lethality	TDP-43 proteinopathy	Histology hallmark	Phenotype		Ref.
					Loss nuclear TDP-43	Ubiquitin aggregate	Cognitive dysfunction	Motor dysfunction	
Mouse	*Tardbp*-deficient	deleted exon 2 and 3 of *Tardbp*	ubiquitous	O	O	NA	NA	NA	[38]
	*Tardbp*^−/−^	gene trap insertion of intron 2 and lead to in-frame fusion	ubiquitous	O	O	NA	NA	NA	[39]
	*Tardbp*^−/−^	Gene trap and insert β-geo after exon 2 of *Tardbp*	ubiquitous	O	O	NA	NA	NA	[40]
	Conditional *Tardbp*-KO	Er-Cre x *Tardbp^F/F^* (floxed exon 3)	ubiquitous	X	O	NA	NA	NA	[41]
	HB9:Cre-*Tardbp^lx/^*^−^	HB9-Cre x *Tardbp^lx^* (floxed exon 2 and 3)	spinal cord motor neuron	X	O	O	NA	O	[42]
	TDP CKO	VAChT-Cre x TDP-43^flox/flox^ (floxed exon 2)	motor neuron	X	O	NA	NA	O	[43]

**Table 4:** Non-mammalian model: wild-type or mutant TDP-43 expression by transgenic animals (NA: non-analysis).

Species	Transgene	expression site (promoter)	TDP-43 proteinopathy						Phenotype	Ref.
			TDP-43+ inclusion	ubiquitin+ and TDP-43+ inclusion	Cytoplasmic TDP-43	Loss nuclear TDP-43	Truncated TDP-43 (35 or 25 kDa)	Phosphorylated TDP-43	Motor dysfunction	
*C. elegans*	TDP-1	ubiquitous (snb1)	NA	NA	X	X	NA	NA	O	[44]
	WT-hTDP-43	ubiquitous (snb1)	NA	X	X	X	NA	NA	O	
	WT-hTDP-43	ubiquitous (snb1)	O	O	X	X	O	X	O	[45]
	A315T-hTDP-43	ubiquitous (snb1)	O	O	X	X	O	O	O	
	G290A-hTDP-43	ubiquitous (snb1)	O	NA	X	X	O	O	O	
	M337V-hTDP-43	ubiquitous (snb1)	O	NA	X	X	O	O	O	
	WT-hTDP-43-YFP	ubiquitous (snb1)	O	NA	X	O	NA	NA	O	[46]
	Q331K-hTDP-43-YFP M337V-hTDP-43-YFP	ubiquitous (snb1)	NA	NA	NA	NA	NA	NA	O	
	C25-hTDP-43-YFP	ubiquitous (snb1)	O	NA	O	NA	NA	NA	O	
	WT-hTDP-43	GABAergic neuron (unc-47)	X	NA	NA	NA	NA	NA	X	[47]
	A315T-hTDP-43	GABAergic neuron (unc-47)	O	NA	NA	NA	NA	NA	O	
Zebrafish	WT-hTDP-43	ubiquitous (CMV)	NA	NA	NA	NA	NA	NA	X	[48]
	A315T-hTDP-43 G384C-hTDP-43 A382T-hTDP-43	ubiquitous (CMV)	NA	NA	NA	NA	NA	NA	O	
	Y220X	ubiquitous	NA	NA	NA	NA	NA	NA	NA	[49]
Drosophila	dTDP-43 WT-hTDP-43 M337V-hTDP-43 Q331K-hTDP-43 209-414 a.a of hTDP-43	sensory neuron	NA	NA	NA	NA	NA	NA	NA	[50]
	WT-hTDP-43-RFP	eye (GMR)	O	X	O	NA	NA	NA	NA	[51]
	T202-hTDP-43-RFP	eye (GMR)	NA	NA	NA	NA	NA	NA	NA	
	WT-hTDP-43-RFP	mushroom body (OK107)	NA	NA	NA	NA	NA	NA	NA	
	WT-hTDP-43-RFP	motor neuron (OK371)	O	X	O	NA	NA	NA	O	
	WT-hTDP-43	eye (GMR)	NA	NA	NA	NA	NA	NA	NA	[52]
	WT-hTDP-43	motor neuron (D42)	O (Rare)	NA	O (Rare)	NA	NA	NA	NA	
	WT-hTDP-43 NES-mut-hTDP-43 NLS-mut-hTDP-43M337V-hTDP-43	eye (GMR)	NA	NA	NA	NA	O	NA	NA	[53]
	WT-hTDP-43	eye (GMR)	NA	NA	NA	NA	NA	NA	NA	[54]
	WT-hTDP-43 Q331K-hTDP-43	motor neuron (D42)	NA	NA	NA	NA	NA	NA	O	
	WT-hTDP-43 A315T-hTDP-43	pan neuronal (elav) or motor neuron (D42)	NA	NA	X	X	NA	NA	O (D42)	[55]
	G287S-hTDP-43 G348C-hTDP-43 A382T-hTDP-43 N390D-hTDP-43	pan neuronal (elav) or motor neuron (D42)	NA	NA	X	X	NA	NA	NA	
	NLS-mut-hTDP-43	pan neuronal (elav) or motor neuron (D42)	NA	NA	O	O	NA	NA	NA	
	CTF-hTDP-43		NA	NA	O	O	NA	NA	O (D42)	
	FFLL-hTDP-43		NA	NA	O^a^	X	NA	NA	O (D42)	
	WT-hTDP-43	eye (GMR) or pan neuronal (elav)	X	NA	O	X	O	O	NA	[56]
	NES-mut-hTDP-43		O	NA	X	X	O	O	NA	
	NLS-mut-hTDP-43		X	NA	O	O	O	O	NA	
	WT-hTDP-43 A315T-hTDP-43	eye (GMR) or motor neuron (D42)	O (aggregate in axon)	NA	O	NA	NA	NA	O (D42)	[57]
	dTDP-43	mushroom body (OK107) or motor neuron (D42)	O	NA	O	NA	NA	NA	O (D42)	[58]
	CTF-hTDP-43	pan neuronal (elav)	O	NA	O	NA	NA	O	NA	[59]
	Mutant CTF of TDP-43 b		O	NA	O	NA	NA	X	NA	
	Mutant CTF of TDP-43^C^		X	NA	O	NA	NA	O	NA	
	WT-TBPH(dTDP-43)	motor neuron (D42)	NA	NA	O	NA	NA	NA	O	[60]
	UAS-dTDP-43-Flag	CCAP neuron (ccap)	O	O (NII)	O	X	NA	NA	NA	[61]
	WT-TBPH(dTDP-43)	pan-neuronal (elav) or upper motor neuron (EB1) or eye (GMR)	X (GMR)	X (GMR)	X (GMR)	X (GMR)	NA	NA	O (elav or EB1)	[62]

a : while with high level of FFLL-hTDP-43;

b : CTF-hTDP-43 (Mutation in S 379, 403, 404, 409, 410 to A);

c : CTF-hTDP-45 (Mutation in S 379, 403, 404, 409, 410 to E).

**Table 5:** Non-mammalian model: TDP-43 knockout model (NA: non-analysis).

Species	Line	Deletion	Deletion site	Embryonic lethality	TDP-43 proteinopathy	Phenotype	Ref.
					Loss nuclear TDP-43	Motor dysfunction	
*C. elegans*	*ok803*	deletion mutant which removes two RNA Recognize Motifs and the nucleare export signal of *TDP-1*	ubiquitous	X	O	O	[63,64]
	*ok781*	deletion mutant which removes two RNA Recognize Motifs and the nucleare export signal of *TDP-1*	ubiquitous	X	NA	O	[63]
Zebrafish	TDP-43 AMO	an AMO sequence complimentary to tranlational start site of *tardbp*	ubiquitous	X	NA	O	[48]
	TDP-43 AMO	*tardbpl* AMO	ubiquitous	X	NA	O	[49]
	*tardbp*^−/−^	genome editing with zinc finger nucleases, which target to *tardbp*	ubiquitous	X	NA	X	[65]
	*tardbpl*^−/−^	genome editing with zinc finger nucleases, which target to *tardbpl*	ubiquitous	X	NA	X	
	*tardbpl*^−/−^ and *tardbp*^−/−^	genome editing with zinc finger nucleases, which target to *tardbp* and *tardbpl*	ubiquitous	X	NA	O	
Drosophila	*TBPH-KO*	imprecise mobilization of *TBPH* transposone by transposase	ubiquitous	X	O	O	[66]
	*TBPH-null*	use P-element mobilization to delete *TBPH*	ubiquitous	O	NA	NA	[67]
	*dTDP^ex26^*	use P-element mobilization to delete *TBPH*	ubiquitous	X	O	O	[58]
	*TBPH^DD100^*, *TBPH^DD96^*	use imprecise P-element mobilization to delete *TBPH*	ubiquitous	X	NA	O	[62]

## References

[1] McMurtray A., Clark D.G., Christine D., Mendez M.F. (2006). Early-onset dementia: Frequency and causes compared to late-onset dementia. Dement. Geriatr. Cogn. Disord.

[2] Neary D., Snowden J.S., Gustafson L., Passant U., Stuss D., Black S., Freedman M., Kertesz A., Robert P.H., Albert M. (1998). Frontotemporal lobar degeneration: A consensus on clinical diagnostic criteria. Neurology.

[3] Sieben A., van Langenhove T., Engelborghs S., Martin J.J., Boon P., Cras P., de Deyn P.P., Santens P., van Broeckhoven C., Cruts M (2012). The genetics and neuropathology of frontotemporal lobar degeneration. Acta Neuropathol.

[4] Al-Chalabi A., Jones A., Troakes C., King A., Al-Sarraj S., van den Berg L.H. (2012). The genetics and neuropathology of amyotrophic lateral sclerosis. Acta Neuropathol.

[5] Neumann M., Sampathu D.M., Kwong L.K., Truax A.C., Micsenyi M.C., Chou T.T., Bruce J., Schuck T., Grossman M., Clark C.M. (2006). Ubiquitinated TDP-43 in frontotemporal lobar degeneration and amyotrophic lateral sclerosis. Science.

[6] Chen-Plotkin A.S., Lee V.M., Trojanowski J.Q. (2010). TAR DNA-binding protein 43 in neurodegenerative disease. Nat. Rev. Neurol.

[7] Yeung C.W., Woo M., Lee K., Greer C.W. (2011). Characterization of the bacterial community structure of Sydney Tar Ponds sediment. Can. J. Microbiol.

[8] Wang H.Y., Wang I.F., Bose J., Shen C.K. (2004). Structural diversity and functional implications of the eukaryotic *TDP* gene family. Genomics.

[9] Wang I.F., Wu L.S., Chang H.Y., Shen C.K. (2008). TDP-43, the signature protein of FTLD-U, is a neuronal activity-responsive factor. J. Neurochem.

[10] Polymenidou M., Lagier-Tourenne C., Hutt K.R., Huelga S.C., Moran J., Liang T.Y., Ling S.C., Sun E., Wancewicz E., Mazur C. (2011). Long pre-mRNA depletion and RNA missplicing contribute to neuronal vulnerability from loss of TDP-43. Nat. Neurosci.

[11] Liu-Yesucevitz L., Bilgutay A., Zhang Y.J., Vanderweyde T., Citro A., Mehta T., Zaarur N., McKee A., Bowser R., Sherman M. (2010). Tar DNA binding protein-43 (TDP-43) associates with stress granules: Analysis of cultured cells and pathological brain tissue. PLoS One.

[12] Bentmann E., Neumann M., Tahirovic S., Rodde R., Dormann D., Haass C (2012). Requirements for stress granule recruitment of fused in sarcoma (FUS) and TAR DNA-binding protein of 43 kDa (TDP-43). J. Biol. Chem.

[13] Cairns N.J., Neumann M., Bigio E.H., Holm I.E., Troost D., Hatanpaa K.J., Foong C., White C.L., Schneider J.A., Kretzschmar H.A. (2007). TDP-43 in familial and sporadic frontotemporal lobar degeneration with ubiquitin inclusions. Am. J. Pathol.

[14] Gitcho M.A., Bigio E.H., Mishra M., Johnson N., Weintraub S., Mesulam M., Rademakers R., Chakraverty S., Cruchaga C., Morris J.C. (2009). TARDBP 3′-UTR variant in autopsy-confirmed frontotemporal lobar degeneration with TDP-43 proteinopathy. Acta Neuropathol.

[15] Wegorzewska I., Bell S., Cairns N.J., Miller T.M., Baloh R.H. (2009). TDP-43 mutant transgenic mice develop features of ALS and frontotemporal lobar degeneration. Proc. Natl. Acad. Sci. USA.

[16] Stallings N.R., Puttaparthi K., Luther C.M., Burns D.K., Elliott J.L. (2010). Progressive motor weakness in transgenic mice expressing human TDP-43. Neurobiol. Dis.

[17] Xu Y.F., Gendron T.F., Zhang Y.J., Lin W.L., D’Alton S., Sheng H., Casey M.C., Tong J., Knight J., Yu X. (2010). Wild-type human TDP-43 expression causes TDP-43 phosphorylation, mitochondrial aggregation, motor deficits, and early mortality in transgenic mice. J. Neurosci.

[18] Xu Y.F., Zhang Y.J., Lin W.L., Cao X., Stetler C., Dickson D.W., Lewis J., Petrucelli L (2011). Expression of mutant *TDP-43* induces neuronal dysfunction in transgenic mice. Mol. Neurodegener.

[19] Arnold E.S., Ling S.C., Huelga S.C., Lagier-Tourenne C., Polymenidou M., Ditsworth D., Kordasiewicz H.B., McAlonis-Downes M., Platoshyn O., Parone P.A. (2013). ALS-linked *TDP-43* mutations produce aberrant RNA splicing and adult-onset motor neuron disease without aggregation or loss of nuclear TDP-43. Proc. Natl. Acad. Sci. USA.

[20] Wils H., Kleinberger G., Janssens J., Pereson S., Joris G., Cuijt I., Smits V., Ceuterick-de Groote C., van Broeckhoven C., Kumar-Singh S (2010). TDP-43 transgenic mice develop spastic paralysis and neuronal inclusions characteristic of ALS and frontotemporal lobar degeneration. Proc. Natl. Acad. Sci. USA.

[21] Shan X., Chiang P.M., Price D.L., Wong P.C. (2010). Altered distributions of Gemini of coiled bodies and mitochondria in motor neurons of TDP-43 transgenic mice. Proc. Natl. Acad. Sci. USA.

[22] Janssens J., Wils H., Kleinberger G., Joris G., Cuijt I., Ceuterick-de Groote C., van Broeckhoven C., Kumar-Singh S (2013). Overexpression of ALS-associated p.M337V human TDP-43 in mice worsens disease features compared to wild-type human TDP-43 mice. Mol. Neurobiol.

[23] Caccamo A., Majumder S., Oddo S (2012). Cognitive decline typical of frontotemporal lobar degeneration in transgenic mice expressing the 25-kDa *C*-terminal fragment of TDP-43. Am. J. Pathol.

[24] Tsai K.J., Yang C.H., Fang Y.H., Cho K.H., Chien W.L., Wang W.T., Wu T.W., Lin C.P., Fu W.M., Shen C.K. (2010). Elevated expression of TDP-43 in the forebrain of mice is sufficient to cause neurological and pathological phenotypes mimicking FTLD-U. J. Exp. Med.

[25] Igaz L.M., Kwong L.K., Lee E.B., Chen-Plotkin A., Swanson E., Unger T., Malunda J., Xu Y., Winton M.J., Trojanowski J.Q. (2011). Dysregulation of the ALS-associated gene TDP-43 leads to neuronal death and degeneration in mice. J. Clin. Invest.

[26] Tian T., Huang C., Tong J., Yang M., Zhou H., Xia X.G. (2011). TDP-43 potentiates alpha-synuclein toxicity to dopaminergic neurons in transgenic mice. Int. J. Biol. Sci.

[27] Swarup V., Phaneuf D., Bareil C., Robertson J., Rouleau G.A., Kriz J., Julien J.P. (2011). Pathological hallmarks of amyotrophic lateral sclerosis/frontotemporal lobar degeneration in transgenic mice produced with TDP-43 genomic fragments. Brain.

[28] Cannon A., Yang B., Knight J., Farnham I.M., Zhang Y., Wuertzer C.A., D’Alton S., Lin W.L., Castanedes-Casey M., Rousseau L. (2012). Neuronal sensitivity to TDP-43 overexpression is dependent on timing of induction. Acta Neuropathol.

[29] Zhou H., Huang C., Chen H., Wang D., Landel C.P., Xia P.Y., Bowser R., Liu Y.J., Xia X.G. (2010). Transgenic rat model of neurodegeneration caused by mutation in the *TDP* gene. PLoS Genet.

[30] Huang C., Tong J., Bi F., Zhou H., Xia X.G. (2012). Mutant *TDP-43* in motor neurons promotes the onset and progression of ALS in rats. J. Clin. Invest.

[31] Uchida A., Sasaguri H., Kimura N., Tajiri M., Ohkubo T., Ono F., Sakaue F., Kanai K., Hirai T., Sano T. (2012). Non-human primate model of amyotrophic lateral sclerosis with cytoplasmic mislocalization of TDP-43. Brain.

[32] Tatom J.B., Wang D.B., Dayton R.D., Skalli O., Hutton M.L., Dickson D.W., Klein R.L. (2009). Mimicking aspects of frontotemporal lobar degeneration and Lou Gehrig’s disease in rats via TDP-43 overexpression. Mol. Ther.

[33] Dayton R.D., Wang D.B., Cain C.D., Schrott L.M., Ramirez J.J., King M.A., Klein R.L. (2012). Frontotemporal lobar degeneration-related proteins induce only subtle memory-related deficits when bilaterally overexpressed in the dorsal hippocampus. Exp. Neurol.

[34] Dayton R.D., Gitcho M.A., Orchard E.A., Wilson J.D., Wang D.B., Cain C.D., Johnson J.A., Zhang Y.J., Petrucelli L., Mathis J.M. (2013). Selective forelimb impairment in rats expressing a pathological TDP-43 25 kDa *C*-terminal fragment to mimic amyotrophic lateral sclerosis. Mol. Ther.

[35] Wang D.B., Dayton R.D., Henning P.P., Cain C.D., Zhao L.R., Schrott L.M., Orchard E.A., Knight D.S., Klein R.L. (2010). Expansive gene transfer in the rat CNS rapidly produces amyotrophic lateral sclerosis relevant sequelae when TDP-43 is overexpressed. Mol. Ther.

[36] Herman A.M., Khandelwal P.J., Rebeck G.W., Moussa C.E. (2012). Wild type *TDP-43* induces neuro-inflammation and alters APP metabolism in lentiviral gene transfer models. Exp. Neurol.

[37] Pesiridis G.S., Tripathy K., Tanik S., Trojanowski J.Q., Lee V.M. (2011). A “two-hit” hypothesis for inclusion formation by carboxyl-terminal fragments of TDP-43 protein linked to RNA depletion and impaired microtubule-dependent transport. J. Biol. Chem.

[38] Wu L.S., Cheng W.C., Hou S.C., Yan Y.T., Jiang S.T., Shen C.K. (2010). TDP-43, a neuro-pathosignature factor, is essential for early mouse embryogenesis. Genesis.

[39] Sephton C.F., Good S.K., Atkin S., Dewey C.M., Mayer P., Herz J., Yu G (2010). TDP-43 is a developmentally regulated protein essential for early embryonic development. J. Biol. Chem.

[40] Kraemer B.C., Schuck T., Wheeler J.M., Robinson L.C., Trojanowski J.Q., Lee V.M., Schellenberg G.D. (2010). Loss of murine TDP-43 disrupts motor function and plays an essential role in embryogenesis. Acta Neuropathol.

[41] Chiang P.M., Ling J., Jeong Y.H., Price D.L., Aja S.M., Wong P.C. (2010). Deletion of TDP-43 down-regulates *Tbc1d1*, a gene linked to obesity, and alters body fat metabolism. Proc. Natl. Acad. Sci. USA.

[42] Wu L.S., Cheng W.C., Shen C.K. (2012). Targeted depletion of TDP-43 expression in the spinal cord motor neurons leads to the development of amyotrophic lateral sclerosis-like phenotypes in mice. J. Biol. Chem.

[43] Iguchi Y., Katsuno M., Niwa J., Takagi S., Ishigaki S., Ikenaka K., Kawai K., Watanabe H., Yamanaka K., Takahashi R. (2013). Loss of TDP-43 causes age-dependent progressive motor neuron degeneration. Brain.

[44] Ash P.E., Zhang Y.J., Roberts C.M., Saldi T., Hutter H., Buratti E., Petrucelli L., Link C.D. (2010). Neurotoxic effects of TDP-43 overexpression in *C. elegans*. Hum. Mol. Genet.

[45] Liachko N.F., Guthrie C.R., Kraemer B.C. (2010). Phosphorylation promotes neurotoxicity in a *Caenorhabditis elegans* model of TDP-43 proteinopathy. J. Neurosci.

[46] Zhang T., Mullane P.C., Periz G., Wang J (2011). TDP-43 neurotoxicity and protein aggregation modulated by heat shock factor and insulin/IGF-1 signaling. Hum. Mol. Genet.

[47] Vaccaro A., Tauffenberger A., Aggad D., Rouleau G., Drapeau P., Parker J.A. (2012). Mutant TDP-43 and FUS cause age-dependent paralysis and neurodegeneration in *C. elegans*. PLoS One.

[48] Kabashi E., Lin L., Tradewell M.L., Dion P.A., Bercier V., Bourgouin P., Rochefort D., Bel Hadj S., Durham H.D., Vande Velde C. (2010). Gain and loss of function of ALS-related mutations of TARDBP (TDP-43) cause motor deficits *in vivo*. Hum. Mol. Genet..

[49] Hewamadduma C.A., Grierson A.J., Ma T.P., Pan L., Moens C.B., Ingham P.W., Ramesh T., Shaw P.J. (2013). Tardbpl splicing rescues motor neuron and axonal development in a mutant tardbp zebrafish. Hum. Mol. Genet.

[50] Lu Y., Ferris J., Gao F.B. (2009). Frontotemporal dementia and amyotrophic lateral sclerosis-associated disease protein TDP-43 promotes dendritic branching. Mol. Brain.

[51] Li Y., Ray P., Rao E.J., Shi C., Guo W., Chen X., Woodruff E.A., Fushimi K., Wu J.Y. (2010). A Drosophila model for TDP-43 proteinopathy. Proc. Natl. Acad. Sci. USA.

[52] Hanson K.A., Kim S.H., Wassarman D.A., Tibbetts R.S. (2010). Ubiquilin modifies TDP-43 toxicity in a Drosophila model of amyotrophic lateral sclerosis (ALS). J. Biol. Chem.

[53] Ritson G.P., Custer S.K., Freibaum B.D., Guinto J.B., Geffel D., Moore J., Tang W., Winton M.J., Neumann M., Trojanowski J.Q. (2010). TDP-43 mediates degeneration in a novel Drosophila model of disease caused by mutations in VCP/p97. J. Neurosci.

[54] Elden A.C., Kim H.J., Hart M.P., Chen-Plotkin A.S., Johnson B.S., Fang X., Armakola M., Geser F., Greene R., Lu M.M. (2010). Ataxin-2 intermediate-length polyglutamine expansions are associated with increased risk for ALS. Nature.

[55] Voigt A., Herholz D., Fiesel F.C., Kaur K., Muller D., Karsten P., Weber S.S., Kahle P.J., Marquardt T., Schulz J.B. (2010). TDP-43-mediated neuron loss *in vivo* requires RNA-binding activity. PLoS One.

[56] Miguel L., Frebourg T., Campion D., Lecourtois M (2011). Both cytoplasmic and nuclear accumulations of the protein are neurotoxic in Drosophila models of TDP-43 proteinopathies. Neurobiol. Dis.

[57] Estes P.S., Boehringer A., Zwick R., Tang J.E., Grigsby B., Zarnescu D.C. (2011). Wild-type and A315T mutant TDP-43 exert differential neurotoxicity in a Drosophila model of ALS. Hum. Mol. Genet.

[58] Lin M.J., Cheng C.W., Shen C.K. (2011). Neuronal function and dysfunction of Drosophila dTDP. PLoS One.

[59] Li H.Y., Yeh P.A., Chiu H.C., Tang C.Y., Tu B.P. (2011). Hyperphosphorylation as a defense mechanism to reduce TDP-43 aggregation. PLoS One.

[60] Hazelett D.J., Chang J.C., Lakeland D.L., Morton D.B. (2012). Comparison of parallel high-throughput RNA sequencing between knockout of TDP-43 and its overexpression reveals primarily nonreciprocal and nonoverlapping gene expression changes in the central nervous system of Drosophila. G3 (Bethesda).

[61] Vanden Broeck L., Naval-Sanchez M., Adachi Y., Diaper D., Dourlen P., Chapuis J., Kleinberger G., Gistelinck M., van Broeckhoven C., Lambert J.C. (2013). TDP-43 loss-of-function causes neuronal loss due to defective steroid receptor-mediated gene program switching in Drosophila. Cell Rep.

[62] Diaper D.C., Adachi Y., Sutcliffe B., Humphrey D.M., Elliott C.J., Stepto A., Ludlow Z.N., Vanden Broeck L., Callaerts P., Dermaut B. (2013). Loss and gain of Drosophila TDP-43 impair synaptic efficacy and motor control leading to age-related neurodegeneration by loss-of-function phenotypes. Hum. Mol. Genet.

[63] Zhang T., Hwang H.Y., Hao H., Talbot C., Wang J (2012). *Caenorhabditis elegans* RNA-processing protein TDP-1 regulates protein homeostasis and life span. J. Biol. Chem.

[64] Vaccaro A., Tauffenberger A., Ash P.E., Carlomagno Y., Petrucelli L., Parker J.A. (2012). TDP-1/TDP-43 regulates stress signaling and age-dependent proteotoxicity in *Caenorhabditis elegans*. PLoS Genet.

[65] Schmid B., Hruscha A., Hogl S., Banzhaf-Strathmann J., Strecker K., van der Zee J., Teucke M., Eimer S., Hegermann J., Kittelmann M. (2013). Loss of ALS-associated TDP-43 in zebrafish causes muscle degeneration, vascular dysfunction, and reduced motor neuron axon outgrowth. Proc. Natl. Acad. Sci. USA.

[66] Feiguin F., Godena V.K., Romano G., D’Ambrogio A., Klima R., Baralle F.E. (2009). Depletion of TDP-43 affects Drosophila motoneurons terminal synapsis and locomotive behavior. FEBS Lett.

[67] Fiesel F.C., Voigt A., Weber S.S., van den Haute C., Waldenmaier A., Gorner K., Walter M., Anderson M.L., Kern J.V., Rasse T.M. (2010). Knockdown of transactive response DNA-binding protein (TDP-43) downregulates histone deacetylase 6. EMBO J.

[68] Tauffenberger A., Vaccaro A., Aulas A., Vande Velde C., Parker J.A. (2012). Glucose delays age-dependent proteotoxicity. Aging Cell.

[69] Vaccaro A., Patten S.A., Ciura S., Maios C., Therrien M., Drapeau P., Kabashi E., Parker J.A. (2012). Methylene blue protects against TDP-43 and FUS neuronal toxicity in *C. elegans* and *D. rerio*. PLoS One.

[70] Audet J.N., Soucy G., Julien J.P. (2012). Methylene blue administration fails to confer neuroprotection in two amyotrophic lateral sclerosis mouse models. Neuroscience.

[71] Vaccaro A., Patten S.A., Aggad D., Julien C., Maios C., Kabashi E., Drapeau P., Parker J.A. (2013). Pharmacological reduction of ER stress protects against TDP-43 neuronal toxicity *in vivo*. Neurobiol. Dis..

[72] Liachko N.F., McMillan P.J., Guthrie C.R., Bird T.D., Leverenz J.B., Kraemer B.C. (2013). CDC7 inhibition blocks pathological TDP-43 phosphorylation and neurodegeneration. Ann. Neurol.

[73] Swarup V., Phaneuf D., Dupre N., Petri S., Strong M., Kriz J., Julien J.P. (2011). Deregulation of TDP-43 in amyotrophic lateral sclerosis triggers nuclear factor kappaB-mediated pathogenic pathways. J. Exp. Med.

[74] Wang I.F., Guo B.S., Liu Y.C., Wu C.C., Yang C.H., Tsai K.J., Shen C.K. (2012). Autophagy activators rescue and alleviate pathogenesis of a mouse model with proteinopathies of the TAR DNA-binding protein 43. Proc. Natl. Acad. Sci. USA.

[75] Wang I.F., Tsai K.J., Shen C.K. (2013). Autophagy activation ameliorates neuronal pathogenesis of FTLD-U mice: A new light for treatment of TARDBP/TDP-43 proteinopathies. Autophagy.

[76] Armstrong G.A., Drapeau P (2013). Calcium channel agonists protect against neuromuscular dysfunction in a genetic model of TDP-43 mutation in ALS. J. Neurosci.

[77] Kanazawa M., Kakita A., Igarashi H., Takahashi T., Kawamura K., Takahashi H., Nakada T., Nishizawa M., Shimohata T (2011). Biochemical and histopathological alterations in TAR DNA-binding protein-43 after acute ischemic stroke in rats. J. Neurochem.

[78] Lavu S., Boss O., Elliott P.J., Lambert P.D. (2008). Sirtuins—Novel therapeutic targets to treat age-associated diseases. Nat. Rev. Drug Discov.

[79] Brooks M (1933). MEthylene blue as antidote for cyanide and carbon monoxide poisoning. J. Am. Med. Assoc.

[80] Schirmer R.H., Coulibaly B., Stich A., Scheiwein M., Merkle H., Eubel J., Becker K., Becher H., Muller O., Zich T. (2003). Methylene blue as an antimalarial agent. Redox Rep.

[81] Kwok E.S., Howes D (2006). Use of methylene blue in sepsis: A systematic review. J. Intensive Care Med.

[82] Wen Y., Li W., Poteet E.C., Xie L., Tan C., Yan L.J., Ju X., Liu R., Qian H., Marvin M.A. (2011). Alternative mitochondrial electron transfer as a novel strategy for neuroprotection. J. Biol. Chem.

[83] Poteet E., Winters A., Yan L.J., Shufelt K., Green K.N., Simpkins J.W., Wen Y., Yang S.H. (2012). Neuroprotective actions of methylene blue and its derivatives. PLoS One.

[84] Zhang H., Tan C.F., Mori F., Tanji K., Kakita A., Takahashi H., Wakabayashi K (2008). TDP-43-immunoreactive neuronal and glial inclusions in the neostriatum in amyotrophic lateral sclerosis with and without dementia. Acta Neuropathol.

[85] Caccamo A., Majumder S., Deng J.J., Bai Y., Thornton F.B., Oddo S (2009). Rapamycin rescues TDP-43 mislocalization and the associated low molecular mass neurofilament instability. J. Biol. Chem.

[86] Davidson Y.S., Raby S., Foulds P.G., Robinson A., Thompson J.C., Sikkink S., Yusuf I., Amin H., DuPlessis D., Troakes C. (2011). TDP-43 pathological changes in early onset familial and sporadic Alzheimer’s disease, late onset Alzheimer’s disease and Down’s syndrome: Association with age, hippocampal sclerosis and clinical phenotype. Acta Neuropathol.

[87] Amador-Ortiz C., Lin W.L., Ahmed Z., Personett D., Davies P., Duara R., Graff-Radford N.R., Hutton M.L., Dickson D.W. (2007). TDP-43 immunoreactivity in hippocampal sclerosis and Alzheimer’s disease. Ann. Neurol.

[88] Arai T., Mackenzie I.R., Hasegawa M., Nonoka T., Niizato K., Tsuchiya K., Iritani S., Onaya M., Akiyama H (2009). Phosphorylated TDP-43 in Alzheimer’s disease and dementia with Lewy bodies. Acta Neuropathol.

[89] Higashi S., Iseki E., Yamamoto R., Minegishi M., Hino H., Fujisawa K., Togo T., Katsuse O., Uchikado H., Furukawa Y. (2007). Concurrence of TDP-43, tau and alpha-synuclein pathology in brains of Alzheimer’s disease and dementia with Lewy bodies. Brain Res.

[90] Rayaprolu S., Fujioka S., Traynor S., Soto-Ortolaza A.I., Petrucelli L., Dickson D.W., Rademakers R., Boylan K.B., Graff-Radford N.R., Uitti R.J. (2013). TARDBP mutations in Parkinson’s disease. Parkinsonism Relat. Disord.

[91] Zhang Y.J., Xu Y.F., Dickey C.A., Buratti E., Baralle F., Bailey R., Pickering-Brown S., Dickson D., Petrucelli L (2007). Progranulin mediates caspase-dependent cleavage of TAR DNA binding protein-43. J. Neurosci.

